# Kousso on the Tape-Worm

**Published:** 1852-08

**Authors:** 


*Kousso on the Tape-Worm.* By J. A. Frydinger, M. D., New Orleans.

In compliance with a promise made to you some time since, I now give you the result of my experience with the new remedy for Tape-worm—Kousso. I have used it in two cases only, which I will relate as briefly as possible.

The first was a young gentleman, about the age of twenty-one years, a resident of this city. He had suffered from it from childhood, and had been treated for it by a number of physicians, both in England and in this country, previous to my acquaintance with him. He states that no treatment heretofore used had afforded him any but partial relief, and at last would mitigate his suffering only for a few days. The entire catalogue of anthelmentics had been thoroughly tried in his case, particularly the terebinthinate preparations, and pushed to an extent, in several instances, to seriously affect his general health, and as he states, in one or two, to endanger his life; still the animal was not destroyed and his sufferings continued.

I have treated him for the last three years, at intervals, with varied results, but invariably gave him some relief for a time; at times he would discharge from a few separate joints to several hundred; at others, portions of the worm measuring in length from three or four inches to seventy or eighty feet; and I may here state, that since I commenced treating him, he has discharged over *a thousand feet of worm.*

In February last it annoyed him very much, and I determined to try the Kousso on him; accordingly, half an ounce was administered in water at bed time, followed in the morning by a seidlitz powder. In a few hours his bowels were moved, but no signs of worm or the Kousso could be discovered in the evacuations. During the following night the Kousso came away bringing with it a large quantity of the worm, broken, and torn, and mangled to such a degree, that it was some time before it could be recognized in the mass of matter. Such portions as could be selected from the mass, of sufficient size and form, worthy of preservation, can be seen at my office, with specimens of entire worm from the same person.

With a view of making it as certain as possible, and to give it a full trial, after the lapse of a week the dose was repeated; but this time it was premised by a dose of castor oil, for the purpose of emptying the bowels, so as to give the remedy entire control of the alimentary canal, and to encounter nothing but the animal itself; but no traces of worm could be observed in the operations of the bowels; nor since the operation of the first dose. The young man is positive that the animal has been entirely destroyed.

The other and last case was also a resident of this city, a lady, about thirty-five years of age. She had passed portions of worm for fifteen or eighteen years. She suffered greatly from indigestion, derangement of the bowels, menses, etc., mostly all her life.

A half ounce of Kousso was given her about the first of March last, which brought away a large amount of worm, broken up as in the former case. In this case no cathartic medicine was given either before or subsequent to the administration of the Kousso.

Her digestion has since greatly improved; her bowels and menses have become regular; and she has gained considerable flesh and color; in a word her general health has greatly improved. She states she never was as well in her life before. So far she has had no return of the symptoms of the worm, and believes she is entirely relieved.

I am not prepared to give a decided opinion as regards the action of the Kousso upon the worm; but you will readily infer from the foregoing (the appearance of the worm as brought away by the remedy) that I am inclined to the opinion that it acts mechanically upon it; analogous to the Dolichos Pruriens on the Lumbricoides.

Upon reflecting on this method, after having made the promise, I came to the conclusion to defer this communication longer than was originally intended, for the reason that I wished some time to elapse, to see whether there would be a return of it in either of the cases, as I desired to give a statement that could, to some extent, be relied on.

Although several months have elapsed since the medicine was given, and no symptoms of the existence of the worm have reappeared, still I am not yet satisfied that they will not return. Should they return in either case, I will advise you of it.

In conclusion, I would say, that although not certain that this article will or can entirely eradicate the worm, and drive it from the system, I am satisfied that it is the most effective remedy I have ever tried in its removal, and I have used all the remedies I ever heard of, and have treated a good many persons suffering from Tape-worm. I would further add, that there is no danger in using it whatever; no unpleasant symptoms occurred in either of the cases; and did not even disqualify them for their ordinary pursuits.—*New Orleans Med. and Surg. Jour.*

